# Differentiating Transient From Persistent Developmental Delays in a Nationwide Infant Cohort

**DOI:** 10.1001/jamanetworkopen.2025.39441

**Published:** 2025-10-27

**Authors:** Yonatan Bilu, Guy Amit, Keren Mayer Lapidot, Noa Gueron-Sela, Nira Kerber, Meytal Avgil Tsadok, Yair Sadaka

**Affiliations:** 1 KI Research Institute, Kfar Malal, Israel; 2 Department of Psychology, Ben-Gurion University of the Negev, Be’er-Sheva, Israel; 3 Negev INSPIRE, Mental Health Institute, Be’er-Sheva, Israel; 4 TIMNA Initiative Big Data Platform, Ministry of Health, Jerusalem, Israel

## Abstract

**Importance:**

Early childhood developmental surveillance is critical for identifying children at risk of developmental delays and ensuring timely intervention. Current well-child surveillance policies are not well designed to distinguish transient delays from persistent ones, potentially leading to unnecessary referrals or missed opportunities for early support.

**Objective:**

To evaluate whether the Tipat Halav Israeli Surveillance (THIS) developmental scale can be leveraged for distinguishing transient from persistent developmental delays and thus augment the precision of early childhood surveillance policies.

**Design, Setting, and Participants:**

This retrospective cohort study used national data from maternal child health clinics in Israel that perform routine developmental surveillance of children aged 0 to 6 years, serving approximately 65% of Israeli children. Participants were individuals born at 37 weeks’ gestation or later in the years 2014 to 2022 and assessed between January 1, 2014, and December 31, 2022, who failed to attain at least 1 developmental milestone between age 9 and 12 months based on their electronic health records. Analysis was done between July 2024 and April 2025.

**Exposure:**

Age-appropriate milestone attainment results at ages 9 to 12 months, along with demographic and birth-related covariates.

**Main Outcome and Measures:**

The primary outcome was ongoing milestone attainment failure at age 12 to 24 months. Accuracy of machine learning models trained to predict the outcome was measured by the area under the receiver operating characteristic curve, while for simpler decision rules, sensitivity and specificity were computed.

**Results:**

In a cohort of 529 797 infants born at 37 weeks’ gestation or later and assessed at age 9 to 12 months, developmental delays were observed in 37 760 (7.1%), among whom 20 862 (55.2%) were male and median gestational age was 39 weeks (IQR, 38-40 weeks). A total of 35 163 (93.1%) were assessed again in their subsequent year of life, among whom persistent delay was suggested for 8802 (25.0%). Machine learning models distinguished transient from persistent delays, with areas under the receiver operating characteristic curve ranging from 0.71 (95% CI, 0.70-0.72) to 0.77 (95% CI, 0.74-0.79). Simply counting the number of developmental domains in which unmet milestones occurred showed good fidelity as well (eg, sensitivity of 0.48 and specificity of 0.81 to identify persistent gross motor delay after early milestone failure in that domain plus 1 other).

**Conclusions and Relevance:**

This cohort study of infants showing early signs of developmental delay suggests that transient delays can be effectively distinguished from persistent ones using routine well-child surveillance data and also provides evidence for the validity of counting domains in which milestones are not attained, which is often used in practice. This suggests an accessible approach for developmental surveillance that may improve outcomes for children worldwide.

## Key Points

**Question:**

Among infants who show early developmental milestone failure at well-child visits, can routine surveillance be leveraged to identify those at risk for a persistent delay?

**Findings:**

In this cohort study of 37 760 infants who exhibited early developmental delay at 9 to 12 months, both machine learning and rule-based approaches showed good fidelity in identifying individuals at risk for persistent developmental delays.

**Meaning:**

The findings suggest that early childhood surveillance can be leveraged to identify children at risk for persistent developmental delay, thereby improving referral accuracy and resource allocation.

## Teaser

This cohort study assesses the accuracy of early childhood development surveillance at identifying risk of persistent developmental delay among infants who fail to attain 1 or more milestones at age 9 to 12 months.

## Introduction

Developmental milestones during the early years of life are important indicators of a child’s overall health and future developmental trajectory.^1^ A significant proportion of children worldwide experience delays in achieving these milestones, which may be early signs of underlying motor, cognitive, linguistic, or social challenges.^2,3,4^ To address this, the Centers for Disease Control and Prevention (CDC)^5^ and the American Academy of Pediatrics^6^ recommend routine early childhood developmental surveillance using milestone attainment norms, such as the CDC’s Developmental Milestones Checklist, though importantly this milestone checklist is not a substitute for a standardized, validated developmental screening tool.^7^ Such tools are widely used in various settings, including by primary caregivers, health care practitioners, daycare centers, and early childhood education environments.^6^

Developmental surveillance has advanced significantly in recent years with evidence-based tools^8^ and evidence-informed methods,^9^ facilitating more accurate detection of delays in achieving developmental milestones. In Israel, developmental surveillance is guided by the Tipat Halav Israeli Surveillance (THIS) developmental scale, a validated tool based on over 4 million developmental assessments in a multicultural population.^10^ It consists of short, age-related milestone checklists with standardized thresholds for normative attainment ages.

A key challenge in addressing delays in milestones achievement following early identification is distinguishing between children who will naturally catch up and those needing specialized assessment and intervention.^9^ Some delays, caused by late maturation, transient hypotonia, or limited environmental exposure, resolve with minimal support, while others signal persistent developmental issues requiring intervention. Mild cases often improve with brief, targeted support, while ongoing difficulties necessitate continuous monitoring by specialists.^11,12,13,14^

Herein, we outline data-driven methods for using the THIS scale to effectively distinguish between children with transient developmental delays and those with more persistent delays, with the aim of facilitating timely and appropriate intervention for all children. To formalize this in a way that aligns with the available data, our study considered children who failed to attain developmental milestones and aimed to identify for whom among them this would continue to be the case at an older age by considering all the available information at the time of the risk likelihood estimation. We consider this as a step toward extended use of the THIS scale as an effective screening tool.

## Methods

This cohort study followed the Strengthening the Reporting of Observational Studies in Epidemiology (STROBE) reporting guideline. Analysis was done between July 2024 and April 2025. All analyses were carried out in accordance with relevant guidelines and regulations. The study protocol was approved by the Soroka University Medical Center institutional ethical committee, which waived the need for informed consent owing to the use of deidentified data.

### Developmental Surveillance in Israel

Developmental surveillance in Israel is routinely conducted from birth to 6 years of age in accordance with national guidelines in approximately 1000 maternal and child health clinics (MCHCs) using a standardized protocol that assesses 59 developmental milestones across 4 domains: personal-social, language, fine motor, and gross motor. Parents are encouraged to visit MCHCs after postpartum hospital discharge and at regular intervals (at ages 1, 3, 6, 9, 12, 18, 24, 36, 48, and 60 months). Attendance rates are particularly high during the first 2 years (approximately 95%), when these visits often align with the national immunization schedule.

During each visit, age-appropriate developmental milestones (“age steps”) are assessed and compared with standardized norms established by the THIS scale (freely available online^15^). Milestone attainment is typically determined through direct observation during the clinic visit, although parental reporting accounts for approximately 10% of assessments. If a milestone is not achieved via observation or parental report, it is recorded as unattained. Children who do not achieve milestones by the age threshold corresponding to the 95th percentile of the national normative range are referred to a pediatrician for assessment of potential developmental delays. Herein we denote such milestone failure as “severe milestone failure.” Notably, the THIS scale also estimates age thresholds at which 90% and 75% of children attain a milestone when the data allow—if 95% of children attain a certain milestone at all ages recorded in the dataset, then these lower thresholds cannot be computed.

This study used data from clinics managed by the Ministry of Health, major municipalities, and Leumit Health Services, which collectively serve about 65% of Israeli children. Comparative analysis with national statistics indicated that the study cohort was fairly demographically representative (eTable 1 in Supplement 1).^10^ Data from MCHC visits are documented in a unified electronic health record (EHR) system. This EHR system integrates sociodemographic and birth-related information with visit-specific records on developmental milestone attainment.

### Study Cohorts and Outcome

Study cohorts were based on children born at 37 weeks’ gestation or later in the years 2014 to 2022 and assessed between January 1, 2014, and December 31, 2022. Our main analysis considered children who were assessed at age 9 to 12 months and severely failed to attain at least 1 of this age step’s milestones (eTable 2 in Supplement 1). Specifically, we considered 4 cohorts defined by the domain of the unattained milestone—language-social (combining these 2 domains), fine motor, gross motor, and any (failing to attain a milestone in any domain). We considered these children as being at risk for developmental delay (in these domains), and the research aim was therefore to identify for whom this risk of delay would be transient and for whom it would be persistent. The language and social domains were combined because social milestones at very early ages are few, and at such ages categorizing a milestone as belonging to one domain rather than the other may be subjective (eg, “vocalizes in dialogue”).

To differentiate between transient and persistent delays, we looked at the children’s assessments at ages 12 to 24 months and identified children with severe milestone failure therein, considering these failures as representative of a persistent delay (eTable 3 in Supplement 1 lists milestones at this age range). In the first 3 cohorts, we considered 2 outcomes—severe failure of a milestone from the domain that defined the cohort (specific persistent delay [SPD]) and severe failure of some milestone (general persistent delay [GPD]). In the fourth cohort (any milestone), we only considered GPD, since no specific domain was associated with it. For example, considering the gross motor cohort (children who severely failed to attain a milestone from the gross motor domain at age 9 to 12 months), children with a GPD outcome were those who severely failed some milestone at age 12 to 24 months, whereas those with an SPD outcome were a subset of children who failed a gross motor milestone at age 12 to 24 months (eFigure 1 in Supplement 1).

Taken together, these definitions implied a longitudinal retrospective cohort study with the following inclusion criteria: (1) year of birth from 2014 to 2022, (2) gestational age of at least 37 weeks, (3) milestone assessments conducted at ages 9 to 12 months and 12 to 24 months, and (4) severe failure of a milestone at age 9 to 12 months. The outcomes examined were SPD and GPD.

### Predictive Models

Machine learning models were trained to predict the outcomes by using milestone attainment information, encoded as developmental surveillance scores (DSS).^7^ In brief, DSS transforms binary milestone attainment results into continuous values between 0 and 4 using the THIS scale and then averages these values over multiple milestones and visits. Hence, each failure is assigned a score reflecting its frequency among the same age population. The greater the proportion of children who attain the milestone at this age, the higher the corresponding DSS. Such scores have previously been shown instrumental in estimating risk for autism spectrum disorder.^16^ Additionally, the machine learning models included demographic, obstetric, and neonatal covariates extracted from the EHR—sex, birth weight, gestational age, birth type (cesarean, instrumental, or spontaneous), and Apgar scores at 1 minute and 5 minutes—along with the mother’s age, marital status, employment status, and maximal attained educational level. Information about ethnicity and socioeconomic status was not included to avoid bias and to make the models generalizable to non-Israeli settings. All these covariates were defined using data that were aggregated up to the time point when the child was 12 months old, with DSS based solely on the first visit at age 9 to 12 months. This emulated risk assessment at age 12 months based on demographic data, birth data, and milestone assessment at the age step of 9 to 12 months.

Modeling was done using gradient-boosting trees, which were trained and evaluated by 5-fold cross-validation, using Python, version 3.9.13 (Python Software Foundation), and XGBoost, version 1.15.0. Model hyperparameters were set as described previously.^16^ These same hyperparameters were used for all cohorts and all outcome prediction evaluations.

### Decision Rules

In addition to the machine learning–based predictive models, we examined simple decision rules to identify children at risk for persistent delays—severe failure in milestones from 2 domains (the 2-domains rule) and severe failure in milestones from all 3 domains (the 3-domains rule). Of note, in the 3 cohorts defined by severe failure in a specific domain, the 2-domains rule is equivalent to saying that a child severely failed a milestone from a domain other than the cohort-defining one. When evaluating these rules, we considered 2 types of measures: (1) sensitivity vs specificity and (2) the fraction of children estimated to be at risk for persistent delay vs the positive predictive value (PPV) (ie, among the children who were identified to be at risk for persistent delay, the fraction that indeed severely failed to attain a milestone at age 12-24 months).

### Sensitivity Analyses

In a sensitivity analyses, we examined the same framework when varying some of the defining parameters. Specifically, we examined the area under the receiver operating characteristic curve (AUC) of models and the sensitivity-specificity tradeoffs of the 2 decision rules with 3 variations of the definitions. In the “later outcome” variation, persistent delay was identified at the age step of 24 to 36 months instead of 12 to 24 months. In the main analysis, developmental delay was defined as failure at an age at which 95% of children attain the milestones; in the “lower failure thresholds” analyses, we tested lower age thresholds of 90%, 75%, and 0% (any failure), and since many milestones have only the 95% threshold defined in the age step of 9 to 12 months, we expanded the milestones used for cohort definition and DSS computation to be 6 to 12 months. In the “all failures cohorts” variation, we considered the cohorts of infants with some milestone attainment failure at age 9 to 12 months (regardless of threshold) and aimed to identify who among them was at risk for a severe (95% threshold) failure at age 12 to 24 months. In this third variation, we added an additional decision rule, which we denoted the “95% rule,” where we identified children who severely failed a milestone in the cohort-defining domain as at risk for persistent delay (specific or general). This rule is similar to the current policy of referral and was relevant only for the 3 cohorts defined by a developmental domain.

### Statistical Analysis

When comparing covariate distribution among the domain-defined cohorts, we used a χ^2^ test of independence and applied Bonferroni correction to the resultant *P* values. Two-sided *P* values of .05 or lower were considered significant.

To evaluate the performance of the main predictive model, the AUC was computed via 5-fold cross-validation; confidence intervals were estimated using bootstrapping (1000 iterations). To evaluate the decision rules, the sensitivity and specificity of each rule were computed. Since this yielded a pair of numbers that were not readily comparable, we also computed the diagnostic odds ratio (DOR),^17^ which integrates the specificity and sensitivity into a single score. To assess whether the difference between 2 sensitivity-specificity pairs was statistically significant, we used the McNemar test for paired nominal data. Analyses were performed using Python statsmodels, version 0.13.2 (Python Software Foundation).^18^

## Results

Of the 529 797 individuals in the dataset born at 37 weeks’ gestation or later and assessed at age 9 to 12 months, 37 760 (7.1%) severely failed to attain at least 1 milestone in any domain and were included in the “any milestone” cohort (16 898 [44.8%] female, 20 862 [55.2%] male; median gestational age, 39 weeks [IQR, 38-40 weeks]). Among them, 15 010 (39.8%) failed to attain a milestone from the language-social domain, 6890 (18.2%) from the gross motor domain, and 21 214 (56.2%) from the fine motor domain (Table 1 and eFigure 1 in Supplement 1).

**Table 1.** Distribution of Covariates Describing a Child’s Birth Measurements and Mother’s Demographics in the Main Cohort Who Severely Failed Any Milestone and Subcohorts Who Severely Failed a Milestone in a Specific Domain at Ages 9 to 12 Months

Covariate	Children, No. (%)			
	Failed language-social milestone (n = 15 010)	Failed gross motor milestone (n = 6890)	Failed fine motor milestone (n = 21 214)	Failed any milestone (n = 37 760)
**Child characteristics**				
Child’s sex				
Female	6457 (43.0)	3630 (52.7)	9139 (43.1)	16 898 (44.8)
Male	8553 (57.0)	3260 (47.3)	12 075 (56.9)	20 862 (55.2)
**Maternal characteristics**				
Employment status				
Missing	4113 (27.4)	1534 (22.3)	6363 (30.0)	10 565 (28.0)
Not working	3387 (22.6)	2297 (33.3)	3319 (15.6)	7580 (20.1)
Student	644 (4.3)	314 (4.6)	739 (3.5)	1476 (3.9)
Working	6866 (45.7)	2745 (39.8)	10 793 (50.9)	18 139 (48.0)
Educational level				
College or higher	4092 (27.3)	1812 (26.3)	7022 (33.1)	11 671 (30.9)
Elementary	401 (2.7)	250 (3.6)	352 (1.7)	828 (2.2)
High school	4300 (28.6)	2624 (38.1)	5563 (26.2)	10 749 (28.5)
Tertiary (eg, vocational)	1597 (10.6)	655 (9.5)	2018 (9.5)	3728 (9.9)
Missing	4620 (30.8)	1549 (22.5)	6259 (29.5)	10 784 (28.6)
Marital status				
Divorced	200 (1.3)	86 (1.2)	345 (1.6)	547 (1.4)
Married	13 175 (87.8)	6086 (88.3)	18 315 (86.3)	32 917 (87.2)
Single	609 (4.1)	260 (3.8)	1082 (5.1)	1732 (4.6)
Widowed	9 (0.1)	3 (0.0)	9 (0.0)	18 (0.0)
Missing	835 (5.6)	372 (5.4)	1169 (5.5)	2052 (5.4)
Other^a^	182 (1.2)	83 (1.2)	294 (1.4)	494 (1.3)
**Birth characteristics**				
Gestational age, wk				
37	1828 (12.2)	958 (13.9)	2611 (12.3)	4555 (12.1)
38	3333 (22.2)	1662 (24.1)	4924 (23.2)	8634 (22.9)
39	4161 (27.7)	1878 (27.3)	5955 (28.1)	10556 (28.0)
40	4017 (26.8)	1736 (25.2)	5417 (25.5)	9916 (26.3)
41	1564 (10.4)	619 (9.0)	2165 (10.2)	3842 (10.2)
42	107 (0.7)	37 (0.5)	142 (0.7)	257 (0.7)
Birth type				
Cesarean	2879 (19.2)	1542 (22.4)	4377 (20.6)	7510 (19.9)
Instrumental	766 (5.1)	368 (5.3)	1323 (6.2)	2171 (5.7)
Spontaneous	10 725 (71.5)	4894 (71.0)	14 677 (69.2)	26 646 (70.6)
Missing	640 (4.3)	86 (1.2)	837 (3.9)	1433 (3.8)
Maternal age, y				
<20	547 (3.6)	366 (5.3)	552 (2.6)	1225 (3.2)
≥40	854 (5.7)	335 (4.9)	1467 (6.9)	2269 (6.0)
Birth weight, kg				
<3	4344 (28.9)	2269 (32.9)	6644 (31.3)	11 299 (29.9)
>4	728 (4.9)	282 (4.1)	875 (4.1)	1691 (4.5)
Apgar score >8^b^				
1 min	13 539 (90.2)	6122 (88.9)	19 002 (89.6)	34 120 (90.4)
5 min	14 235 (94.8)	6543 (95.0)	20 131 (94.9)	35 969 (95.3)

^a^ Marital status was documented as “other” in the electronic health record.

^b^ Apgar scores were available for 97.8% of children in the dataset.

The 4 cohorts displayed similar distributions of birth-related covariates but somewhat different distributions of demographic covariates. In particular, the gross motor cohort stood out as different from the others. For example, in this cohort, only 3260 infants (47.3%) were male, compared with 8553 (57.0%) in the language-social, 12 075 (56.9%) in the fine motor, and 20 862 (55.2%) in the any milestone cohorts (*P* < .001 for each comparison). Similarly, a greater proportion of infants in the gross motor cohort were born by cesarean delivery (1542 [22.4%] vs 2879 [19.2%] in the language-social, 4377 [20.6%] in the fine motor, and 7510 [19.9%] in the any milestone cohorts; all *P* < .001), a smaller proportion had mothers who were working (2745 [39.8%] vs 6866 [45.7%] in the language-social, 10 793 [50.1%] in the fine motor, and 18 139 [48.0%] in the any milestone cohorts; all *P* < .001), and a greater proportion had mothers with high school–level education (2624 [38.1%] vs 4300 [28.6%] in the language-social, 5563 [26.2%] in the fine motor, and 10 749 [28.5%] in the any milestone cohorts; all *P* < .001).

A total of 35 163 children (93.1%) were assessed again at age 12 to 24 months. As described in Table 2, across the 4 cohorts, 90.9% to 95.0% of the individuals evaluated at age 9 to 12 months were evaluated at ages 12 to 24 months for at least 1 language-social or gross motor milestone and 73.7% to 80.4% were evaluated for the fine motor milestone (there was only 1 fine motor milestone in these age steps). Occurrence rate of the GPD outcome in individuals assessed at 12 to 24 months ranged from 8802 cases (23.3%) in the any milestone cohort to 2111 cases (30.6%) in the gross motor cohort, and the rate of the SPD outcome ranged from 423 cases (2.0%) in the fine motor cohort to 1539 cases (22.3%) in the gross motor cohort.

**Table 2.** Statistics of Child Assessment Results at Age 12 to 24 Months, Which Define the Outcomes

Milestone domain	Children^a^			
	Failed language-social milestone at 9-12 mo (n = 15 010)	Failed gross motor milestone at 9-12 mo (n = 6890)	Failed fine motor milestone at 9-12 mo (n = 21 214)	Failed any milestone at 9-12 mo (n = 37 760)
Total assessments, No.	526 035	519 966	526 495	529 797
Language-social				
Evaluated	13 683 (91.2)	6545 (95.0)	19 784 (93.3)	35 158 (93.1)
Failed	3234 (21.5)	1294 (18.8)	3695 (17.4)	6163 (16.3)
Age at failure, mean (IQR), mo	17.18 (13.71-19.07)	17.35 (14.20-19.13)	17.32 (14.12-19.07)	17.15 (13.58-19.00)
Gross motor				
Evaluated	13 649 (90.9)	6541 (94.9)	19 738 (93.0)	35 087 (92.9)
Failed	1852 (12.3)	1539 (22.3)	2977 (14.0)	4676 (12.4)
Age at failure, mean (IQR), mo	18.58 (18.15-19.50)	18.37 (18.15-19.43)	18.52 (18.18-19.36)	18.43 (18.15-19.30)
Fine motor				
Evaluated	11 060 (73.7)	5537 (80.4)	16 402 (77.3)	29 167 (77.2)
Failed	394 (2.6)	180 (2.6)	423 (2.0)	706 (1.9)
Age at failure, mean (IQR), mo	21.86 (21.02-22.49)	21.83 (21.07-22.41)	21.85 (21.01-22.57)	21.84 (21.01-22.49)
Any				
Evaluated	13 685 (91.2)	6548 (95.0)	19 787 (93.3)	35 163 (93.1)
Failed	4001 (26.7)	2111 (30.6)	5190 (24.5)	8802 (23.3)
Age at failure, mean (IQR), mo	17.70 (15.39-19.36)	17.89 (17.08-19.35)	17.83 (16.78-19.30)	17.74 (15.78-19.27)

^a^ Data are presented as number (percentage) of children unless otherwise indicated.

**Figure 1.**
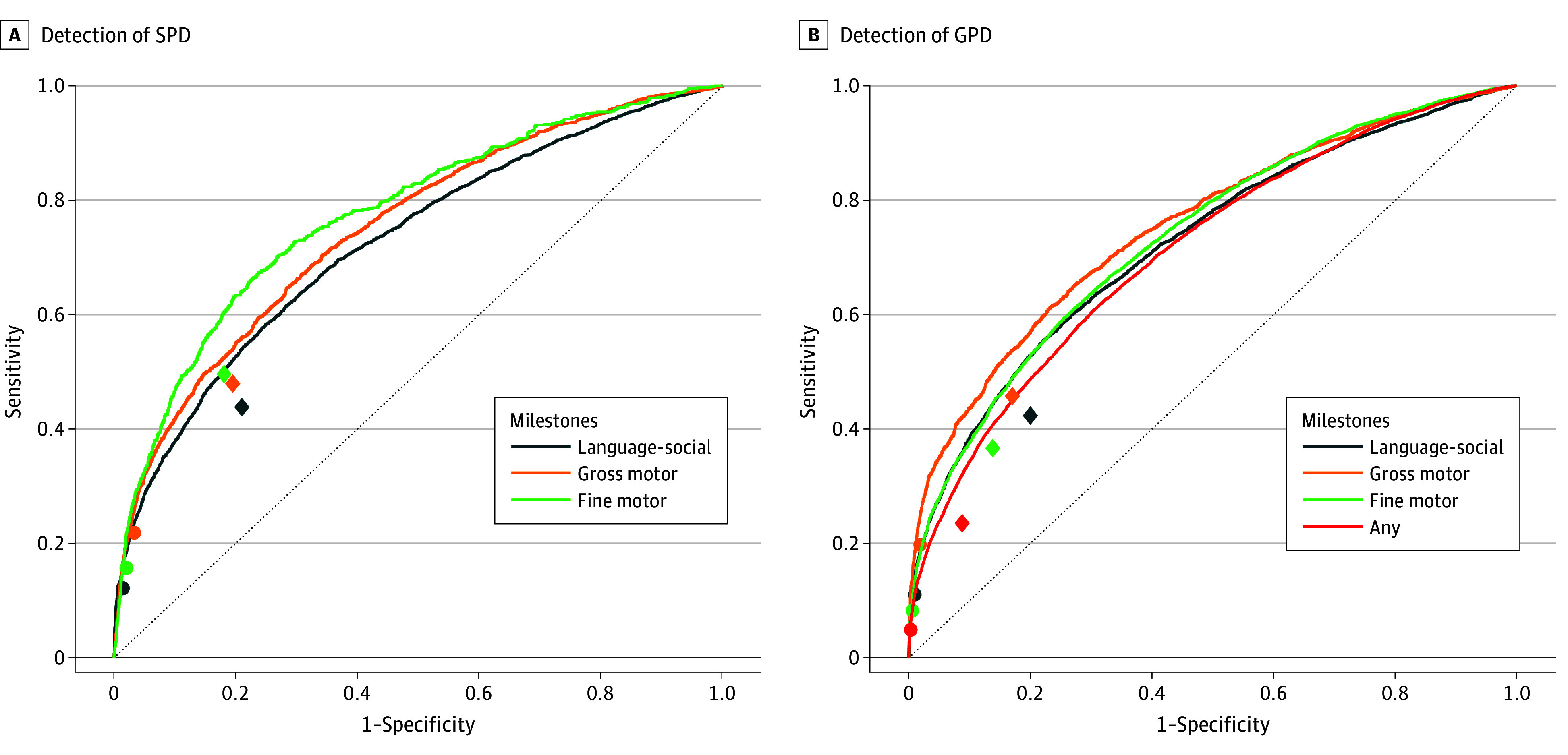
Receiver Operating Characteristic (ROC) Curves for Identification of Specific Persistent Delay (SPD) and General Persistent Delay (GPD) in Developmental Milestones The dotted line represents a random estimation. Diamonds and circles depict the sensitivity and specificity of the 2-domains and 3-domains rules, respectively.

Figure 1 depicts the AUCs of the models aiming to identify GPD between ages 12 and 24 months for the 4 cohorts and SPD for the 3 domain-defined cohorts. For GPD, the AUCs were 0.71 (95% CI, 0.70-0.72) in the any milestone cohort, 0.73 (95% CI, 0.72-0.74) in the language-social cohort, 0.74 (95% CI, 0.73-0.74) in the fine motor cohort, and 0.75 (95% CI, 0.74-0.76) in the gross motor cohort, and for SPD they were 0.73 (95% CI, 0.72-0.74) in the language-social cohort, 0.77 (95% CI, 0.74-0.79) in the fine motor cohort, and 0.75 (95% CI, 0.73-0.76) in the gross motor cohort. Feature importance scores for the respective models are depicted in eFigure 2 in Supplement 1, suggesting that the model’s decision was usually strongly influenced by the developmental scores in the other domains.

The sensitivity and specificity of the 2-domains and 3-domains rules are also depicted in Figure 1. As evident in the figure, both rules were better than a random guess but had lower sensitivity than the machine learning model at the same value of specificity. This difference in sensitivity was statistically significant by McNemar test in nearly all cases (the exception was detection of SPD in the fine motor cohort, where the outcome was rare—423 children [2.0%]). For example, in the gross motor cohort, using the 2-domains rule to identify SPD yielded a specificity of 0.81 and sensitivity of 0.48. At this level of specificity, the sensitivity of the model was 0.54 (*P* = .002).

**Figure 2.**
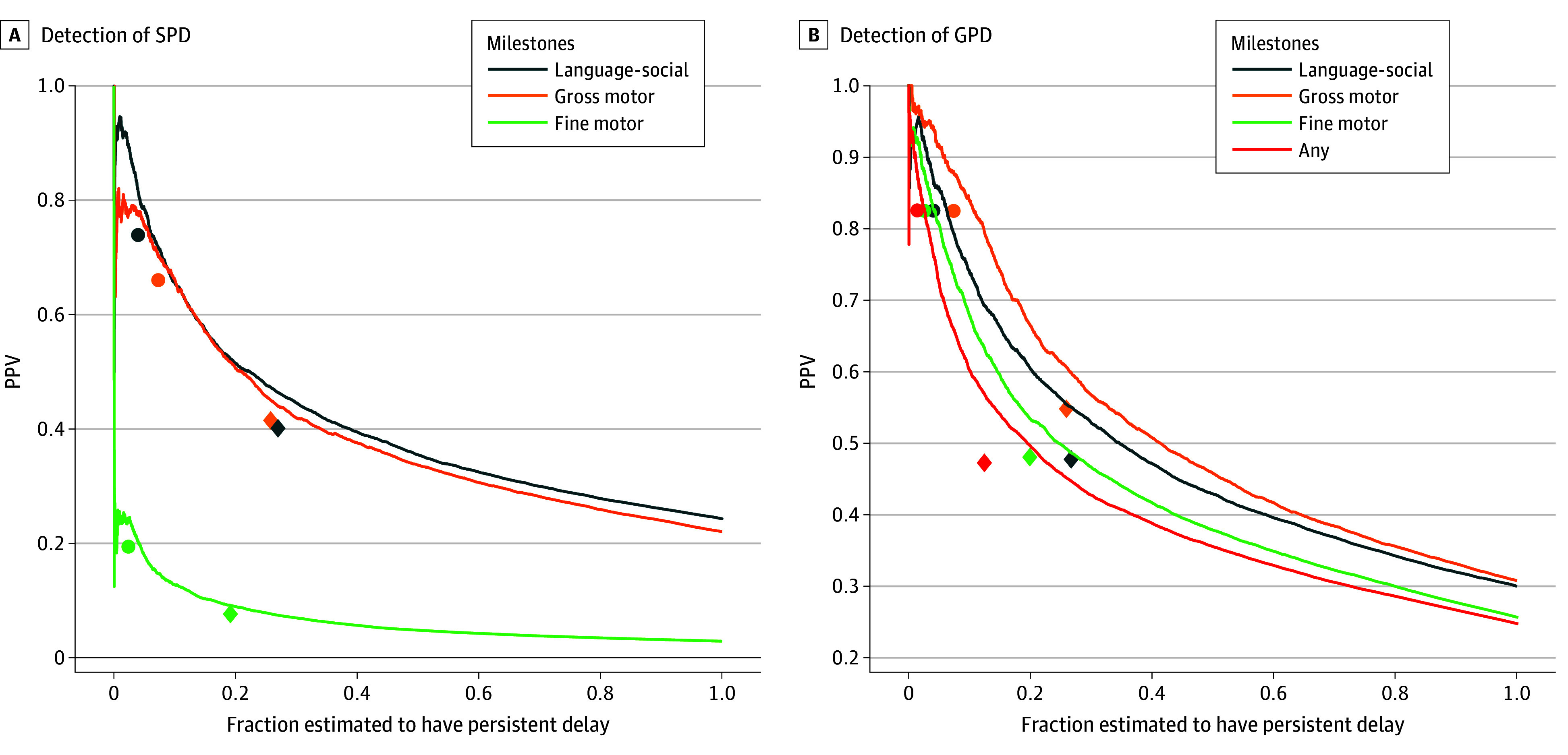
Positive Predictive Value (PPV) vs Fraction at Risk Tradeoffs are depicted between estimation threshold and PPV for models predicting specific persistent delay (SPD) and general persistent delay (GPD) in milestone attainment. Diamonds and circles depict the sensitivity and specificity of the 2-domains and 3-domains rules, respectively.

Figure 2 depicts the tradeoff between the fraction of children estimated to be at risk of persistent delay and the PPV. The 2-domains rule identified 12.2% to 25.7% of children as being at risk, while the 3-domains rule identified 1.5% to 8.1%. For the same quota of predictions, the model had a higher rate of correct predictions (PPV), but in some cases (eg, when identifying a failure in a fine motor milestone), this difference was minor.

### Sensitivity Analyses

Results of the 3 sensitivity analyses (Table 3) were by and large similar to those of the main analysis. In the later-outcome analysis, where we aimed to identify persistent delay by looking at the age step of 24 to 36 months, the AUCs of the models predicting the GPD outcome ranged from 0.70 (95% CI, 0.69-0.71) (any milestone) to 0.76 (95% CI, 0.74-0.78) (gross motor), and for the SPD outcome the AUCs ranged from 0.69 (95% CI, 0.66-0.72) (language-social) to 0.79 (95% CI, 0.77-0.81) (gross motor). No results are provided for fine motor SPD because at the age step of 24 to 36 months, there is only 1 fine motor milestone (“copy lines”), for which the 95% threshold is not defined in the THIS scale (in the data used to construct the scale, at no age do 95% of children attain this milestone). As in the main analysis, the 2-domains rule yielded a sensitivity range of 0.41 to 0.56 and specificity range of 0.76 to 0.91, while the 3-domains rule identified a small number of children, yielding low sensitivity but high specificity of up to 0.99.

**Table 3.** Results of Sensitivity Analyses

Analysis and measure	Domain						
	General persistent delay				Specific persistent delay		
	Language-social	Gross motor	Fine motor	Any	Language-social	Gross motor	Fine motor
**Later outcome**							
AUC (95% CI)	0.72 (0.70-0.74)	0.76 (0.74-0.78)	0.74 (0.73-0.75)	0.70 (0.69-0.71)	0.69 (0.66-0.72)	0.79 (0.77-0.81)	NA
2-Domains rule							
Sensitivity/specificity	0.50/0.79	0.52/0.82	0.42/0.85	0.28/0.91	0.41/0.76	0.56/0.82	NA
DOR	3.78	5.01	4.03	3.75	2.20	5.65	
Rate/PPV^a^	0.25/0.28	0.24/0.37	0.19/0.29	0.11/0.28	0.25/0.06	0.23/0.34	NA
3-Domains rule							NA
Sensitivity/specificity	0.16/0.99	0.27/0.97	0.12/0.99	0.07/0.99	0.10/0.97	0.29/0.97	NA
DOR	12.98	11.95	13.56	14.67	3.42	13.14	
Rate/PPV^a^	0.04/0.64	0.07/0.64	0.02/0.64	0.01/0.64	0.04/0.11	0.07/0.61	NA
**All failures cohort**							
AUC (95% CI)	0.70 (0.70-0.71)	0.69 (0.69-0.70)	0.73 (0.72-0.73)	0.69 (0.68-0.69)	0.70 (0.70-0.71)	0.69 (0.68-0.69)	0.75 (0.73-0.77)
2-Domains rule							
Sensitivity/specificity	0.26/0.90	0.25/0.91	0.28/0.90	0.07/0.99	0.27/0.89	0.25/0.90	0.40/0.87
DOR	3.03	3.33	3.62	5.91	2.93	3.00	4.61
Rate/PPV^a^	0.13/0.34	0.12/0.33	0.14/0.43	0.02/0.47	0.13/0.25	0.12/0.21	0.13/0.06
3-Domains rule							
Sensitivity/specificity	0.04/1.00	0.06/0.99	0.05/1.00	0.02/1.00	0.05/1.00	0.07/0.99	0.10/0.99
DOR	11.74	7.92	14.51	32.31	10.03	6.86	9.74
Rate/PPV^a^	0.01/0.70	0.02/0.58	0.01/0.79	0.00/0.84	0.01/0.57	0.02/0.41	0.01/0.15
95% Rule							
Sensitivity/specificity	0.24/0.88	0.10/0.96	0.53/0.60	NA	0.27/0.88	0.12/0.96	0.56/0.58
DOR	2.34	2.81	1.70	NA	2.68	3.18	1.76
Rate/PPV^a^	0.14/0.29	0.05/0.32	0.42/0.26	NA	0.14/0.24	0.05/0.24	0.42/0.03
**Lower failure thresholds**							
No threshold							
AUC (95% CI)	0.66 (0.65-0.66)	0.68 (0.67-0.68)	0.70 (0.69-0.70)	0.67 (0.67-0.67)	0.65 (0.65-0.65)	0.67 (0.67-0.68)	0.68 (0.68-0.69)
2-Domains rule							
Sensitivity/specificity	0.58/0.54	0.49/0.72	0.81/0.39	0.38/0.78	0.57/0.51	0.51/0.62	0.85/0.28
DOR	1.64	2.54	2.73	2.15	1.40	1.68	2.20
Rate/PPV^a^	0.55/0.77	0.42/0.75	0.75/0.77	0.33/0.75	0.55/0.72	0.42/0.32	0.74/0.19
3-Domains rule							
Sensitivity/specificity	0.17/0.92	0.14/0.95	0.36/0.83	0.09/0.97	0.17/0.91	0.18/0.92	0.46/0.73
DOR	2.28	3.46	2.87	3.22	1.94	2.41	2.29
Rate/PPV^a^	0.14/0.84	0.11/0.84	0.31/0.84	0.07/0.84	0.14/0.80	0.11/0.43	0.30/0.25
75% threshold							
AUC (95% CI)	0.65 (0.65-0.66)	0.68 (0.67-0.68)	0.70 (0.70-0.71)	0.66 (0.65-0.66)	0.64 (0.64-0.65)	0.66 (0.66-0.67)	0.69 (0.68-0.70)
2-Domains rule							
Sensitivity/specificity	0.33/0.80	0.48/0.76	0.63/0.61	0.27/0.85	0.33/0.78	0.48/0.67	0.69/0.50
DOR	1.96	2.85	2.62	2.11	1.73	1.90	2.24
Rate/PPV^a^	0.28/0.71	0.39/0.77	0.53/0.70	0.22/0.71	0.28/0.64	0.39/0.49	0.52/0.18
3-Domains rule							
Sensitivity/specificity	0.04/0.99	0.16/0.96	0.09/0.98	0.03/0.99	0.04/0.99	0.19/0.93	0.16/0.96
DOR	4.81	4.60	4.98	4.98	3.31	3.42	4.11
Rate/PPV^a^	0.03/0.87	0.12/0.87	0.06/0.87	0.02/0.87	0.03/0.79	0.12/0.66	0.06/0.36
90% threshold							
AUC (95% CI)	0.70 (0.70-0.71)	0.70 (0.69-0.72)	0.73 (0.72-0.73)	0.71 (0.70-0.71)	0.69 (0.68-0.70)	0.71 (0.69-0.72)	0.69 (0.68-0.70)
2-Domains rule							
Sensitivity/specificity	0.42/0.78	0.43/0.79	0.31/0.86	0.21/0.91	0.44/0.75	0.44/0.76	0.37/0.83
DOR	2.57	2.85	2.94	2.69	2.36	2.58	2.97
Rate/PPV^a^	0.30/0.58	0.33/0.71	0.20/0.59	0.14/0.59	0.30/0.41	0.33/0.62	0.19/0.26
3-Domains rule							
Sensitivity/specificity	0.07/0.99	0.17/0.97	0.05/1.00	0.03/1.00	0.09/0.99	0.19/0.96	0.08/0.99
DOR	9.47	6.01	10.60	10.50	6.49	5.38	7.90
Rate/PPV^a^	0.03/0.86	0.11/0.86	0.02/0.86	0.01/0.86	0.03/0.70	0.11/0.80	0.02/0.53
95% threshold							
AUC (95% CI)	0.73 (0.72-0.74)	0.75 (0.74-0.76)	0.74 (0.73-0.75)	0.71 (0.71-0.72)	0.74 (0.73-0.75)	0.75 (0.73-0.76)	0.77 (0.74-0.79)
2-Domains rule							
Sensitivity/specificity	0.42/0.81	0.45/0.84	0.37/0.86	0.23/0.91	0.43/0.80	0.48/0.81	0.49/0.82
DOR	3.05	4.26	3.57	3.22	3.04	3.88	4.32
Rate/PPV^a^	0.26/0.47	0.26/0.57	0.20/0.48	0.12/0.47	0.26/0.40	0.26/0.44	0.19/0.07
3-Domains rule							
Sensitivity/specificity	0.11/0.99	0.21/0.98	0.09/0.99	0.05/1.00	0.12/0.99	0.23/0.96	0.15/0.98
DOR	14.00	13.45	15.86	16.28	10.80	7.95	8.13
Rate/PPV^a^	0.04/0.84	0.08/0.84	0.03/0.84	0.02/0.84	0.04/0.75	0.08/0.66	0.02/0.16
**Main analysis**							
AUC (95% CI)	0.73 (0.72-0.74)	0.75 (0.74-0.76)	0.74 (0.73-0.74)	0.71 (0.70-0.72)	0.73 (0.72-0.74)	0.75 (0.73-0.76)	0.77 (0.74-0.79)
2-Domains rule							
Sensitivity/specificity	0.42/0.81	0.45/0.84	0.37/0.86	0.23/0.91	0.43/0.80	0.48/0.81	0.49/0.82
DOR	3.05	4.26	3.57	3.22	3.04	3.88	4.32
Rate/PPV^a^	0.26/0.47	0.26/0.57	0.20/0.48	0.12/0.47	0.26/0.40	0.26/0.44	0.19/0.07
3-Domains rule							
Sensitivity/specificity	0.11/0.99	0.21/0.98	0.09/0.99	0.05/1.00	0.12/0.99	0.23/0.96	0.15/0.98
DOR	14.00	13.45	15.86	16.28	10.80	7.95	8.13
Rate/PPV^a^	0.04/0.84	0.08/0.84	0.03/0.84	0.02/0.84	0.04/0.75	0.08/0.66	0.02/0.16

Abbreviations: AUC, area under the receiver operating characteristic curve; DOR, diagnostic odds ratio; NA, not applicable; PPV, positive predictive value.

^a^ Rate indicates the fraction of children identified as being at risk.

The lower-failure-thresholds analyses suggested that estimation was somewhat more accurate when SPD and GPD were defined using the 95% rule. For example, the AUC was in the range of 0.64 (95% CI, 0.64-0.65) to 0.70 (95% CI, 0.70-0.71) when using the 75% threshold (or any failure) and 0.69 (95% CI, 0.68-0.70) to 0.73 (95% CI, 0.72-0.73) when using the 90% threshold, compared with 0.71 (95% CI 0.71-0.72) to 0.77 (95% CI 0.74-0.79) when using the 95% threshold as in the main analysis (although in this analysis, the DSS was computed over a larger set of milestones—for ages 6-12 months). This trend was also evident when examining the DOR values for each decision rule, as they increased with the threshold in nearly all of the cohorts for both SPD and GPD.

In the all-failures-cohort sensitivity analysis, prediction models achieved AUCs in the range of 0.69 (95% CI, 0.68-0.69) to 0.75 (95% CI, 0.73-0.77); the 2-domains rule tended to yield lower sensitivity (approximately 0.25 for most cases) and higher specificity (approximately 0.90) in the sensitivity analysis than in the main analysis, and similarly, the 3-domains rule yielded sensitivity of 0.10 or below at nearly perfect specificity. We also compared the 95% rule with the 2-domains rule. In the language-social cohort, compared with the 2-domains rule, the 95% rule identified a similar fraction of children as at risk (0.13 vs 0.14, respectively), yet while the PPV for SPD was similar (0.25 vs 0.24, respectively), for GPD it was lower (0.29 for 95% rule vs 0.34 for 2-domains rule). Similarly, in the gross motor cohort, the 95% rule identified the fraction of children at risk as only 0.05, compared with 0.12 by the 2-domains rule, with PPV being roughly the same for SPD (0.24 for 95% rule vs 0.21 for 2-domains rule) and for GPD (0.32 for 95% rule vs 0.33 for 2-domains rule).

## Discussion

Early childhood developmental surveillance is a crucial component of pediatric care, enabling the timely identification of children at risk for developmental delays. However, distinguishing between transient and persistent delays remains a challenge, affecting resource allocation and intervention strategies.^6^ This study addressed this challenge by using a large, multicultural national dataset assessed with the THIS developmental scale and both traditional milestone-based evaluations and predictive modeling to enhance early identification efforts.

The findings suggest that the THIS scale can be leveraged to identify, among children manifesting early signs of developmental delays by the end of their first year of life, those for whom delays will persist during the second and third years. The prediction models, integrating developmental scores with demographic and birth data, achieved good accuracy (AUC approximately 0.75), while simpler decision rules, counting the number of developmental domains with unattained milestones, provided a feasible and more interpretable alternative in contexts where advanced modeling is impractical. Of note, severe failure in milestones from 3 domains successfully identified a small subset of children at high risk with minimal false-positive results, supporting integration of the 3-domains rule into current policies without imposing undue burden on the health care system. To our knowledge, our analysis offers the first quantitative evidence that an intuitive rule already used informally by many clinicians across a wide range of milestone-based monitoring tools does indeed perform reliably, thereby lending an evidence base to everyday practice. These findings align with previous research emphasizing the need for evidence-based developmental surveillance tools^9^ and screening mechanisms that optimize sensitivity and specificity.

A primary contribution of this study is the suggested approach to differentiate between transient developmental delays, typically resulting from minor maturational variations or environmental factors, and persistent delays that may indicate underlying neurodevelopmental conditions. This distinction is essential, as research has shown that children with mild, transient delays frequently catch up to their peers without extensive intervention.^19^ Conversely, children with persistent delays benefit significantly from structured early interventions, as evidenced by studies on targeted support programs in both high-income and low-resource settings.^13,14,20,21^ This differentiation therefore allows for more precise early referrals and follow-up care, augmenting intervention for children at greater risk.

Developmental surveillance is applicable in multiple settings: (1) in daycare centers, where staff can use it to monitor children’s strengths and developmental challenges; (2) by primary caregivers who can track their child’s progress and communicate concerns with health care practitioners, fostering greater parental involvement in early identification efforts; and (3) by health care professionals, including physiotherapists, speech-language therapists, and occupational therapists, who can effectively use developmental surveillance for children referred to their clinics, enabling developmental assessments across multiple domains rather than only the one in which they specialize.

By incorporating decision rules, such as identifying 2 or 3 severe milestone failures, or leveraging predictive models, surveillance can be enhanced in all these settings. This approach is particularly valuable in resource-limited settings, where optimizing specialist referrals helps reduce unnecessary evaluations and ensures that children with persistent developmental concerns receive timely and appropriate support.

### Limitations

Despite its strengths, this study has limitations. While the THIS scale was developed based on over 4 million developmental assessments of diverse, multicultural children, its applicability outside Israel is currently limited and requires further validation. Comparative studies, such as evaluations of the CDC’s “Learn the Signs. Act Early” program and US Head Start initiatives,^5,9^ suggest that cultural and environmental factors influence milestone attainment, necessitating localized adaptations of the THIS framework.^10^ Hence, it would be more appropriate to adapt the framework suggested herein as a blueprint for building a predictive model rather than using it as is in a different setting.

Another challenge lies in equating developmental delay with severe milestone attainment failure. While these factors are strongly correlated, they are not synonymous. Consequently, the numerical results in this study should be interpreted cautiously, as they identify severe milestone failure rather than formal developmental delay diagnoses. Future research should examine whether integrating additional risk factors, longitudinal assessments, and behavioral indicators could enhance identification accuracy and intervention efficacy.

## Conclusions

In this cohort study, we highlighted the potential of the THIS developmental scale as an effective screening tool for child development, bridging the gap between developmental surveillance and targeted intervention. The findings suggest that incorporating structured milestone assessments and predictive models can improve early identification efforts, ensuring efficient use of health care resources while enhancing developmental outcomes. As global efforts continue to refine early childhood screening frameworks, integrating scalable, evidence-based tools can contribute to more equitable and effective developmental monitoring worldwide.

## Notes

Supplement 1. **eFigure 1.** Sketch of Cohorts and Outcomes **eFigure 2.** Feature Importance From XGBoost Modeling **eTable 1.** Distribution of Socioeconomic Clusters as Defined by the Israeli Bureau of Statistics in the Dataset **eTable 2.** Milestones Assessed at Age 9 to 12 Months, Their Domain, and the Age at Which Failure Is Considered Severe **eTable 3.** Milestones Assessed at Ages 12 to 24 Months (Age Step 12-18 Months and Age Step 18-24 Months), Their Domain, and the Age at Which Failure Is Considered Severe

Supplement 2.
Data Sharing Statement
