# Supplementary material for: Differentiating Transient From Persistent Developmental Delays in a Nationwide Infant Cohort
Source: JAMA Netw Open. 2025 Oct 27;8(10):e2539441. doi: 10.1001/jamanetworkopen.2025.39441 (PMC12559967; doi:10.1001/jamanetworkopen.2025.39441)
Supplement: Supplement 1. — eFigure 1. Sketch of Cohorts and Outcomes eFigure 2. Feature Importance From XGBoost Modeling eTable 1. Distribution of Socioeconomic Clusters as Defined by the Israeli Bureau of Statistics in the Dataset eTable 2. Milestones Assessed at Age 9 to 12 Months, Their Domain, and the Age at Which Failure Is Considered Severe eTable 3. Milestones Assessed at Ages 12 to 24 Months (Age Step 12-18 Months and Age Step 18-24 Months), Their Domain, and the Age at Which Failure Is Considered Severe [file jamanetwopen-e2539441-s001.pdf]

Supplementary Online Content

Bilu Y, Amit G, Mayer Lapidot K, et al. Differentiating transient from persistent developmental delays in a nationwide infant cohort. *JAMA Netw Open*. 2025;8(10):e2539441. doi:10.1001/jamanetworkopen.2025.39441

eFigure 1. Sketch of Cohorts and Outcomes

eFigure 2. Feature Importance From XGBoost Modeling

eTable 1. Distribution of Socioeconomic Clusters as Defined by the Israeli Bureau of Statistics in the Dataset

eTable 2. Milestones Assessed at Age 9 to 12 Months, Their Domain, and the Age at Which Failure Is Considered Severe

eTable 3. Milestones Assessed at Ages 12 to 24 Months (Age Step 12-18 Months and Age Step 18-24 Months), Their Domain, and the Age at Which Failure Is Considered Severe

This supplementary material has been provided by the authors to give readers additional information about their work.

© 2025 Bilu Y et al. *JAMA Network Open*.

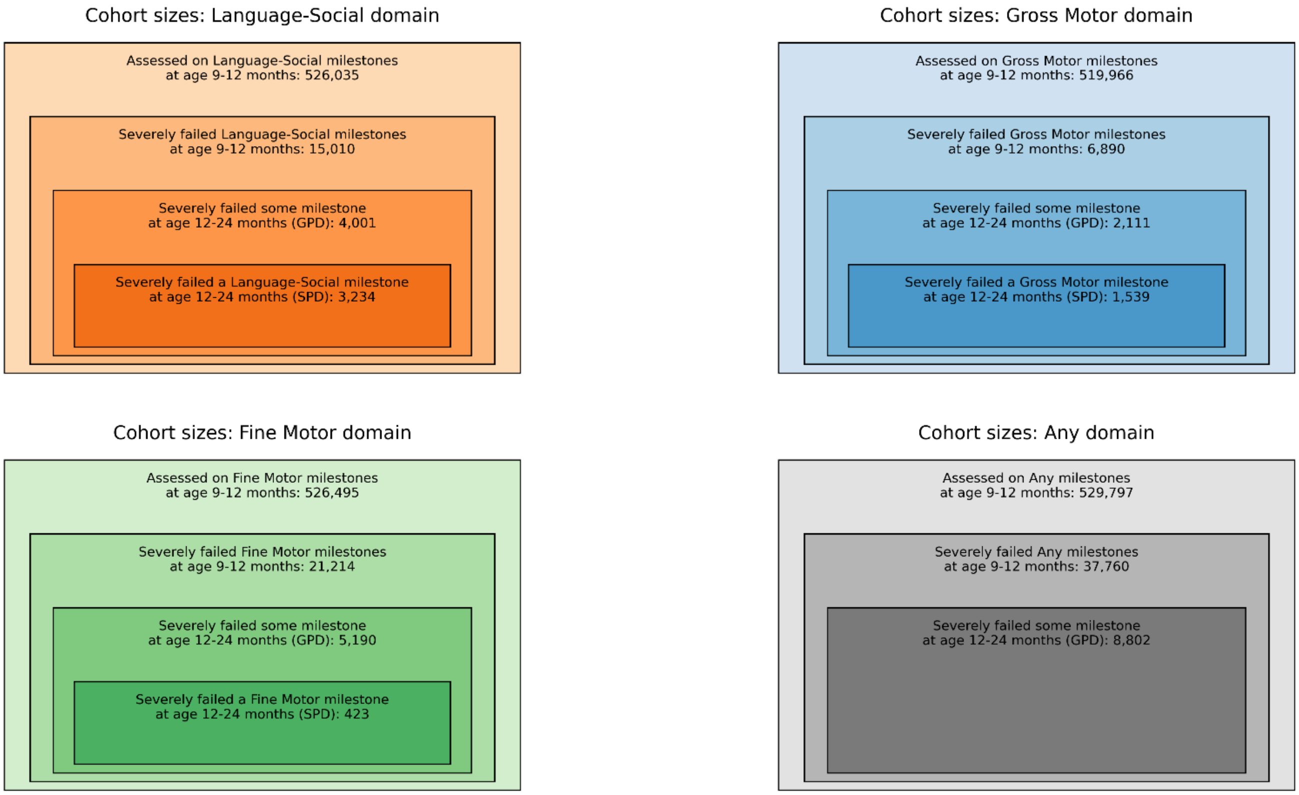

eFigure 1: Sketch of cohorts and outcomes

© 2025 Bilu Y et al. *JAMA Network Open*.

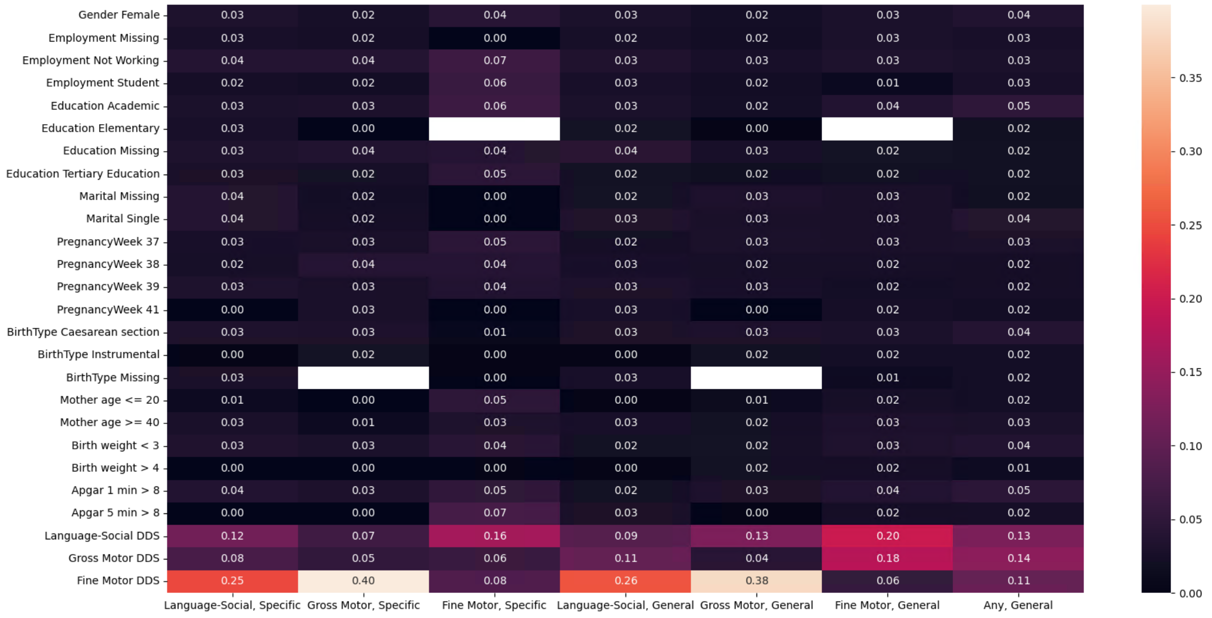

eFigure 2: Feature importance from XGBoost modeling. Scores represent the number of times a feature is used to split the data across all trees. Birth weight is listed in kg. Academic education refers to college level education or higher. Note that these values convey the importance of the features towards the model's prediction, and can not be interpreted directly as measuring the risk for persistent delay associated with them.

© 2025 Bilu Y et al. *JAMA Network Open*.

SES Cluster	Fraction in dataset
1.0	0.14
2.0	0.16
3.0	0.11
4.0	0.06
5.0	0.11
6.0	0.07
7.0	0.10
8.0	0.05
9.0	0.03
10.0	0.02
Missing	0.15

eTable 1: Distribution of socioeconomic clusters as defined by the Israeli Bureau of Statistics, in the dataset. Note that clusters are not deciles, and differ in size.

© 2025 Bilu Y et al. *JAMA Network Open*.

Milestone	Severe failure age (months)	Domain
**crawls**	9	Gross Motor
**gets to sit without support**	12.4	Gross Motor
**taps two objects playfully**	8.8	Fine Motor
**feeds self**	9.1	Fine Motor
**uses thumb-fingers grasp**	10.3	Fine Motor
**vocalizes in a dialogue**	9.1	Language-Social
**responds when addressed by name**	9.1	Language-Social
**understands simple instructions**	11.5	Language-Social
**imitating gesture and movements**	12	Language-Social
**says one word or pronounces meaningful sounds**	12.4	Language-Social

eTable 2: Milestones assessed at age 9-12 months, their domain, and the age at which failure is considered severe. Note that in three cases the latter age is 12 months or higher, and hence failure will never be considered severe.

© 2025 Bilu Y et al. *JAMA Network Open*.

Milestone	Severe failure age (months)	Domain
**builds a tower of cubes**	20.52	Fine Motor
**pulls to stand**	13.58	Gross Motor
**walks with assistance**	13.7	Gross Motor
**walks without assistance**	18.13	Gross Motor
**climbs upstairs with assistance**	18.13	Gross Motor
**makes eye contact during play**	12.13	Language-Social
**expresses will vocally**	12.13	Language-Social
**points at familiar objects**	15.31	Language-Social
**says 2-3 words**	16.54	Language-Social
**eats independently**	18.13	Language-Social
**initiating interactions**	18.13	Language-Social
**functional play**	18.13	Language-Social
**recognizes one body part**	18.81	Language-Social
**gives a kiss**	24.05	Language-Social
**vocabulary > 10 words**	25.87	Language-Social

eTable 3: Milestones assessed at ages 12-24 months (age step 12-18 months and age step 18-24 months), their domain, and the age at which failure is considered severe.

© 2025 Bilu Y et al. *JAMA Network Open*.
