# Supplementary material for: Differentiating Transient From Persistent Developmental Delays in a Nationwide Infant Cohort
Source: JAMA Netw Open. 2025 Oct 27;8(10):e2539441. doi: 10.1001/jamanetworkopen.2025.39441 (PMC12559967; doi:10.1001/jamanetworkopen.2025.39441)
Supplement: Supplement 2. — Data Sharing Statement [file jamanetwopen-e2539441-s002.pdf]

Data Sharing Statement

Bilu. Differentiating Transient From Persistent Developmental Delays in a Nationwide Infant Cohort. *JAMA Netw Open*. Published October 27, 2025.
doi:10.1001/jamanetworkopen.2025.39441

Data
Data available: No

Additional Information
Explanation for why data not available: Data is not allowed to be shared
